# Systemic Transplantation of Adult Multipotent Stem Cells Functionally Rejuvenates Aged Articular Cartilage

**DOI:** 10.14336/AD.2020.1118

**Published:** 2021-06-01

**Authors:** Seth D. Thompson, Rajeswari Pichika, Richard L. Lieber, G.R. Scott Budinger, Mitra Lavasani

**Affiliations:** ^1^Shirley Ryan AbilityLab, Chicago, Illinois, USA.; ^2^Department of Physical Medicine and Rehabilitation, Northwestern University, Chicago, Illinois, USA.; ^3^Northwestern University Interdepartmental Neuroscience (NUIN) Graduate Program, Northwestern University, Chicago, Illinois, USA.; ^4^Division of Pulmonary and Critical Care Medicine, Department of Medicine, Northwestern University, Chicago, Illinois, USA

**Keywords:** aging, articular cartilage, adult stem cells, transplantation, regenerative medicine, gait

## Abstract

Osteoarthritis (OA) is the most common and debilitating joint disease of advanced age and has no universally effective therapy. Here, we demonstrate that systemic transplantation of adult multipotent muscle-derived stem/progenitor cells (MDSPCs)—isolated from young mice—rejuvenates the knee articular cartilage (AC) of naturally aged mice. This intervention reduced expression of pro-inflammatory cytokines (*Tnf* and *Il1a*) and catabolic matrix-degrading proteinases (*Mmp3* and *Mmp13*) in aged cartilage. Treatment with young MDSPCs also increased expression of pro-regenerative (*Col2a1* and *Acan*) and prolongevity genes (*Pot1b*), including those associated with chondrocyte proliferation and differentiation, cartilage growth, and telomere protection. Indeed, the AC of MDSPC-treated mice exhibited reduced age-related histological pathologies. Importantly, the reduced mobility and arthritis-related gait dysfunctions of aged mice were also ameliorated by this treatment. Together, our findings demonstrate the rejuvenating effects of systemic transplantation of young MDSPCs on aging AC—at the molecular, tissue, and functional levels. This suggests that MDSPCs, or their secreted factors, may represent a novel therapy that can increase mobility and function in aged or OA patients.

Aging is the primary risk factor and predictor of articular cartilage (AC) degeneration, which leads to osteoarthritis (OA). The declining ability of aging AC to repair and regenerate is manifested by an increase in inflammatory mediators, matrix degradation, and chondrocyte alterations [1]. Autologous cell therapies based on chondrocyte implantation, matrix-induced chondrogenesis, and transplantation of mesenchymal stem cells have not been successful in older adults [2]. Hence, there is an urgent need for an effective treatment for age-related cartilage degeneration.

We previously discovered that multipotent adult stem cells—muscle-derived stem/progenitor cells (MDSPCs)—isolated from young mice and injected intraperitoneally into mouse models of progeria, delayed the onset of aging-related diseases, doubled lifespan, and improved tissue regeneration [3]. These results strongly suggest that MDSPCs exert therapeutic effects on the aged microenvironment. Here, we report molecular, tissue, and functional outcomes of systemic MDSPC transplantation on aged AC.

## MATERIALS AND METHODS

### MDSPC isolation

Young wild-type (WT) MDSPCs were isolated from the hindlimb skeletal muscle of 21-day-old female mice via a modified preplate technique [4]. MDSPCs were cultured and expanded for transplantation in proliferation medium (PM): Hi-glucose DMEM supplemented with 10% horse serum, 10% fetal-bovine serum (FBS), 1% penicillin-streptomycin (all from Invitrogen), and 0.5% chick embryo extract (CEE, Accurate Chemical). Cells were cultured in collagen type I (Sigma-Aldrich)-coated flasks.

### MDSPC transplantation

All animal experiments were performed with the approval of the Northwestern University Institutional Animal Care and Use Committee. Female WT C57BL/6 mice 20 months of age were obtained from the National Institute of Aging (NIA). The mice were fed ad libitum a gamma-irradiated standard chow. Phosphate buffered saline (PBS) or MDSPCs suspended in PBS were transplanted via intraperitoneal (IP) administration into 22-month-old mice at 2 x 10^5^ MDSPCs per gram body-weight. Mice were sacrificed two months following transplantation at 24 months of age; the right hindlimbs were harvested for histopathological analysis and the left knee joints were used for gene expression analysis.

### RNA isolation and qPCR

To measure mRNA expression, total RNA was extracted from AC isolated from paraformaldehyde (PFA)-fixed knee joints using a RNeasy® mini kit for formalin-fixed, paraffin-embedded (FFPE) tissue (Qiagen) according to manufacturer’s protocol. RNA quality was validated using an Eppendorf Bio-Spectrophotometer, and 100 ng of total RNA was reverse-transcribed according to the manufacture’s protocol using iScript Advanced cDNA synthesis kit (Bio-Rad Laboratories). Pre-amplification of the primers was carried out according to the manufacturer’s protocol using a Pre-amplification kit (Bio-Rad Laboratories). The pre-amplified cDNA was diluted and used for analysis of gene expression changes in 10 μL reactions using SYBR green advanced master mix kit (Bio-Rad Laboratories) and the gene of interest primer pairs. Data were analyzed with the ΔCt method and gene expression was normalized to average expression of *Gapdh* and *Hmbg1*. Primers for the genes of interest were obtained from Bio-Rad Laboratories (Table 1).

**Table 1:** Primer list.

Gene name	Common name	Catalog identification
*Acan*	Aggrecan	qMmuCED0046843
*Adamts5*	a-disintegrin and metalloproteinase with thrombospondin motif 5	qMmuCED0045481
*Bgn*	Biglycan	qMmuCED0046901
*Col1a1*	Collagen type 1 alpha 1	qMuCED0044222
*Col2a1*	Collagen type 2 alpa 1	qMuCID0006546
*Col10a1*	Collagen type 10 alpa 1	qMuCID0008115
*Gadd45a*	Growth arrest and DNA damage inducible alpha	qMmuCED0001074
*Gapdh*	Glyceraldehyde 3-phosphate dehydrogenase	qMmuCED0027497
*Gpx4*	Glutathione peroxidase 4	qMmuCED0001062
*Il1a*	Interleukin 1 alpha	qMmuCID005637
*Mmp3*	Matrix metalloproteinase 3	qMmuCED0049170
*Mmp13*	Matrix metalloproteinase 13	qMmuCED0050490
*Pot1b*	Protection of telomeres 1b	qMmuCED0045942
*Sod1*	Superoxide dismutase 1	qMmuCID0026086
*Tnf*	Tumor necrosis factor	qMmuCED004141
*Vcan*	Versican	qMmuCED0046650

### Histology

Right hind limbs were fixed in PFA for 2 days, stored in PBS at 4^°^C overnight, and then paraffin embedded. Sections were cut to 4 µm, collected at 100 µm intervals, and stained with Safranin O (Saf-O)-Fast Green as described previously [5] to evaluate proteoglycan content and pathological changes such as cartilage degradation.

### Histomorphometry

Histomorphometric analyses were performed using NIS-Elements software (Nikon, AR 5.11.03). The Saf-O+ area was measured impartially by the NIS software using identical thresholding parameters between all images (pixels falling below the threshold intensity of 20 were uncounted) by detecting the total red (Saf-O+) pixel area. The total cartilage area was manually selected with border detection assistance using the NIS software’s thresholding tools. Saf-O+ chondrocytes were manually counted for each femoral condyle and tibial plateau and graphed as average Saf-O+ chondrocytes and number of Saf-O+ chondrocytes per mm^2^ of total AC. Chondrocytes were considered Saf-O+ if they were surrounded by red-stained matrix.

### Gait analysis

All measurements were performed at Northwestern University’s Behavioral Phenotyping Core one-month post-transplantation with an Institutional Animal Care and Use Committee approved animal protocol. Walking speed and gait analysis were assessed using a DigiGait Imaging System (Mouse Specifics Inc.). Briefly, the mice were tested separately within the motorized treadmill chamber at speeds of 10 cm/s, 17 cm/s, and 24 cm/s. Completion of each speed was accomplished when the mouse was able to run uninterrupted for a minimum of 4 s (the amount of time required for DigiGait software to capture sufficient data for analysis) on two separate trials. Each mouse was provided a minimum of five attempts to complete each speed. The percent of successful trials at each speed was recorded as running ability. The stride length, swing duration, and paw area gait parameters were obtained from the data generated using the DigiGait software. Aged mice that did not complete a single trial at a given speed, i.e., those with the greatest functional defects, were therefore unable to generate gait data for analysis. These gait parameters, relevant to aging-related diseases, were chosen in advance to eliminate selection bias from the 20+ gait parameters generated by the DigiGait software.

### Statistics

Statistical analyses were carried out using the SigmaPlot (Jandel Scientific, v14.0) software package. The two-tailed unpaired Welch’s unequal variance *t*-test, two-tailed Student’s *t*-test, or the Mann-Whitney rank sum test were used where appropriate for direct comparison(s) between treatment and control groups. A one-tailed Student’s *t*-test was used to detect functional improvements following MDSPC transplantation. All values are expressed as the mean ± SEM., and *p* < 0.05 was considered statistically significant

## RESULTS

### Articular cartilage of naturally aged mice shows aging-related imbalances associated with local catabolic and anabolic activity

**Figure 1.**
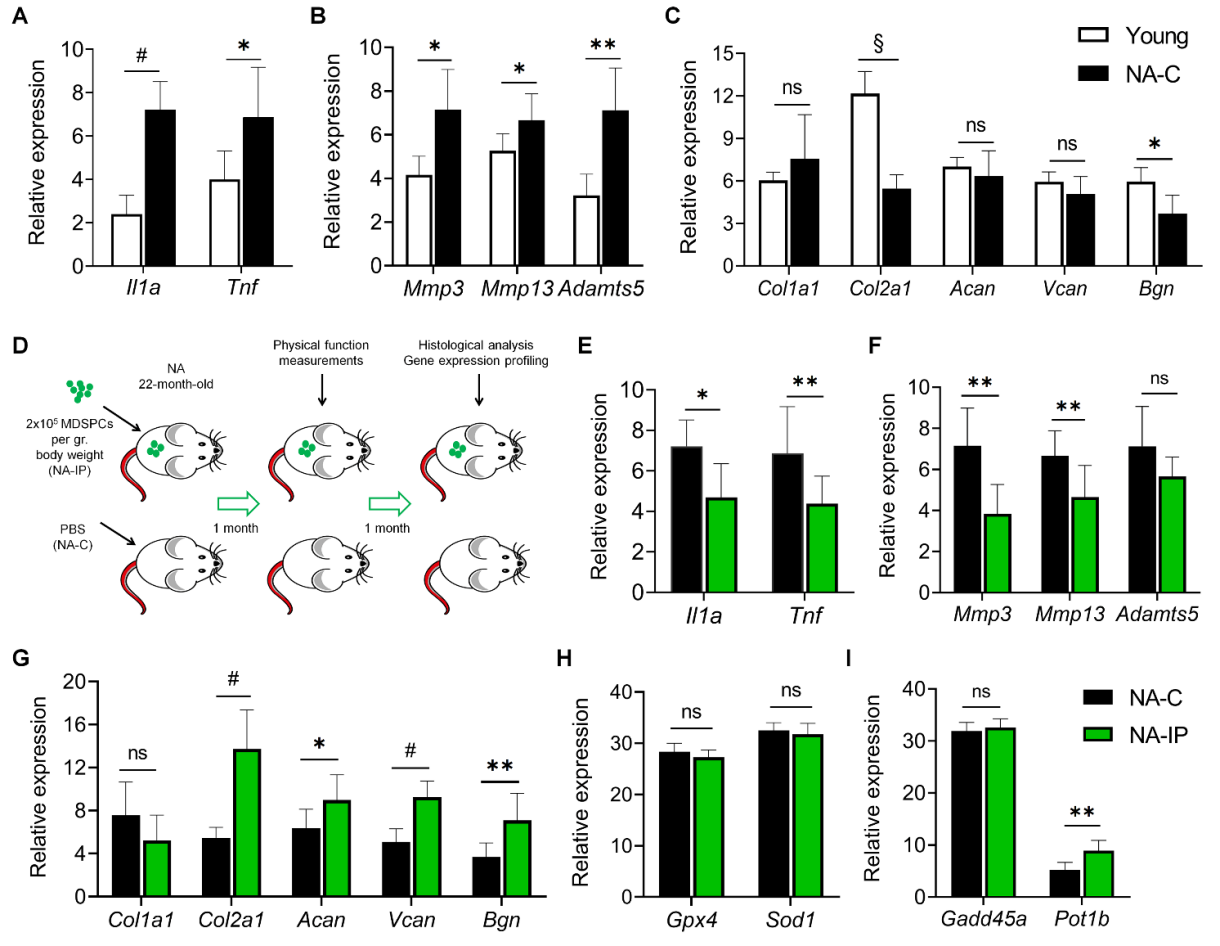
Gene expression profiles in articular cartilage (AC). AC from 5-6-month-old (Young; *n* = 4) and 24-month-old naturally aged control (NA-C; *n* = 7) mice were analyzed by qRT-PCR for expression levels of (A) pro-inflammatory markers, (B) extracellular matrix (ECM) proteinases, and (C) extracellular components. (D) Schematic of cell transplantation experimental design. mRNA expression levels in mice intraperitoneally injected with PBS (NA-C) or muscle-derived stem/progenitor cells (MDSPC) (NA-IP, *n* = 9) were analyzed for (E) pro-inflammatory and SASP markers, (F) ECM proteinases, (G) extracellular components, and genes associated with (H) oxidative stress, (I) growth arrest, and DNA damage responses. Data are presented as mean ± SEM. *p < 0.05, **p < 0.01, ^#^*p* < 0.0001, ^§^*p* < 0.00001, ns: not significant using two-tailed, unpaired Student’s *t*-test or Welch’s unequal variance *t*-test.

To identify changes in the AC of naturally aged (NA) mice, we compared gene expression in PBS-injected NA control (NA-C; 24 months old) to young mice (5-6 months old). Expression of pro-inflammatory cytokine and senescence-associated secretory phenotype (SASP) genes, *Il1a* and *Tnf*—which promote age-related pathologies associated with OA and enhance extracellular matrix (ECM) proteinases [6]—was significantly upregulated in NA-C mice compared to young mice (Fig. 1A). NA-C mice also exhibited a prominent increase in expression of catabolic factors that drive ECM degradation and cleave collagen type II and aggrecans (Fig. 1B), specifically, *Mmp3, Mmp13*, and *Adamts5* [7]. The expression of genes responsible for cartilage tensile strength and tissue repair (*Col2a1*) and cartilage growth (*Bgn*) [8] were also significantly decreased in NA-C mice compared to young (Fig. 1C).

### Systemic transplantation of young MDSPCs ameliorates articular cartilage homeostasis in naturally aged mice

We next examined whether systemic transplantation of young MDSPCs—isolated from 3-week-old mice—ameliorates these OA-like changes. In NA mice intraperitoneally injected with MDSPCs (NA-IP) (Fig. 1D), *Il1a* and *Tnf*, were significantly downregulated in the AC compared to NA-C mice (Fig. 1E). Expression of *Mmp3* and *Mmp13* were also significantly decreased, with *Adamts5* exhibiting a strong, though not statistically significant (*p* = 0.065), decrease (Fig. 1F). Moreover, compared to NA-C mice, NA-IP mice showed significantly increased expression of genes encoding factors involved in cartilage tensile strength and tissue repair (*Col2a1*), resilience (*Acan*), chondrocyte proliferation and differentiation (*Vcan*), and growth (*Bgn*) [8] (Fig. 1G). Similar to previous reports, we did not observe a difference between groups in mRNA expression of *Col1a1* [9]. Aging chondrocytes exhibit senescent phenotypes, including telomere shortening [10]. Notably, expression of the telomere protection and pro-longevity gene [11], *Pot1b*, was significantly increased in NA-IP mice. However, expression levels of other stress response and DNA damage genes—including *Gpx4*, *Sod1*, or *Gadd45a—*remained unchanged (Fig. 1H-I).

### Systemic transplantation of young MDSPCs rejuvenates articular cartilage histology and improves mobility in aged mice

**Figure 2.**
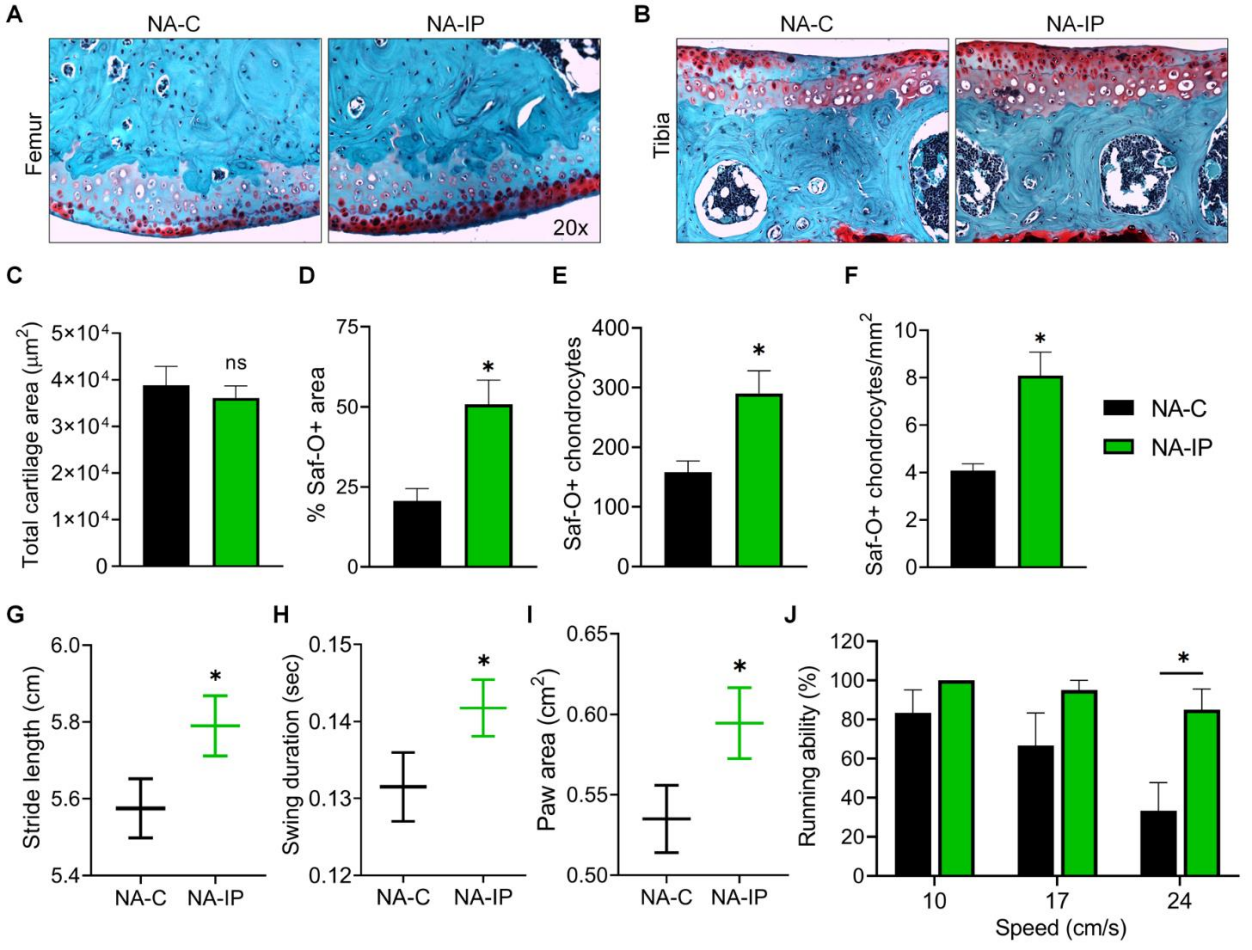
Systemic transplantation of young MDSPCs rejuvenates articular cartilage (AC) and improves mobility and gait in aged mice. Representative 20x magnification images of Safranin-O (Saf-O) stained (A) femoral condyle and (B) tibial plateau from PBS (NA-C; *n* = 3) and MDSPC (NA-IP; *n* = 4) mice two months post intraperitoneal injection. Quantitative histomorphometric analyses of (C) total AC area, (D) percent of Saf-O+ AC area, (E) Saf-O+ chondrocyte number, and (F) Saf-O+ chondrocyte density. Quantifications of gait metrics (G) stride length, (H) swing duration, and (I) paw area. (J) Percentage of NA-C (*n* = 8) or NA-IP (*n* = 10) mice able to run on the DigiGait treadmill at different speeds, one month after transplantation. Data are presented as mean ± SEM. *p < 0.05 with two-tailed for histological data and one-tailed for functional data, using unpaired Student’s *t*-test.

To determine the effects of MDSPC treatment on AC histopathology, we stained the knee joints with Saf-O to detect ECM proteoglycans, which are progressively lost during aging. The femoral condyle (Fig. 2A) and tibial plateau (Fig. 2B) of the AC revealed that while total cartilage area was similar (Fig. 2C), 51% of cartilage in NA-IP mice was Saf-O+ compared to just 21% in NA-C mice (Fig. 2D). NA-IP mice also had greater Saf-O+ chondrocyte numbers (Fig. 2E) and density (Fig. 2F). To identify the functional impact from molecular- and tissue-level rejuvenation, gait parameters were measured one month after MDSPC transplantation using DigiGait. MDSPC treatment significantly increased stride length (Fig. 2G), swing duration (Fig. 2H), and paw area (Fig. 2I) compared to controls. These metrics decline in animal models of age-related movement disorders including arthritis, chronic joint pain, Parkinson’s disease, and diabetic peripheral neuropathy [12-15]. NA-IP mice were also significantly more capable of running at higher speeds (Fig. 2J) than NA-C mice.

## DISCUSSION

Together, our findings demonstrate, for the first time, the rejuvenating effects of systemic transplantation of young MDSPCs on aged AC. This treatment reduced pro-inflammatory factors and ECM proteinases while increasing anabolic factors and proteoglycan content, thereby reversing age-associated degradation. Thus, overcoming these local negative regulators resulted in AC regeneration and significant functional improvements in gait metrics and speed. The mechanism(s) underlying rejuvenation remain speculative and are the main focus of our ongoing studies. However, these results reinforce the paracrine mechanism suggested by our previous work in which systemic transplantation of young MDSPCs significantly extended the lifespan and healthspan of progeroid mice [3]. We hypothesize that MDSPCs orchestrate their pro-regenerative effects by inhibiting AC degeneration and regulating chondrocyte viability and responsiveness to extrinsic factors, which may restore the AC catabolic/anabolic equilibrium in aged hosts to a more “youthful” state. Other possible mechanisms include telomere protection and modulation of the inflammatory SASP cascade. A limitation of this study includes controls not being transplanted with a biologic material, as done in our previous study [3] which demonstrated the necessity of young MDSPCs. It is also possible that due to the systemic nature of this cell therapy, the functional improvements are multifactorial, involving AC, muscle, nerve, and/or bone. Furthermore, translation from murine models to human OA therapies can be difficult and additional safety studies for cell transplantation will need to be performed. However, application of MDSPCs and/or their secreted factors may represent an attractive intervention for aged and OA patients to rejuvenate cartilage and increase mobility.
